# Transcriptomic coordination at hepatic steatosis indicates robust immune cell engagement prior to inflammation

**DOI:** 10.1186/s12864-021-07784-y

**Published:** 2021-06-16

**Authors:** Youwen Zhang, Ioulia Chatzistamou, Hippokratis Kiaris

**Affiliations:** 1 Department of Drug Discovery and Biomedical Sciences, College of Pharmacy, University of South Carolina, CLS 713, 715 Sumter Str., Columbia, SC 29208-3402 USA; 2 Department of Pathology, Microbiology and Immunology, School of Medicine, University of South Carolina, Columbia, SC USA; 3 Peromyscus Genetic Stock Center, University of South Carolina, Columbia, SC USA

**Keywords:** Liver, Correlation network, Pathogenesis, Peromyscus, Transcriptional coordination

## Abstract

**Background:**

Deregulation in lipid metabolism leads to the onset of hepatic steatosis while at subsequent stages of disease development, the induction of inflammation, marks the transition of steatosis to non-alcoholic steatohepatitis. While differential gene expression unveils individual genes that are deregulated at different stages of disease development, how the whole transcriptome is deregulated in steatosis remains unclear.

**Methods:**

Using outbred deer mice fed with high fat as a model, we assessed the correlation of each transcript with every other transcript in the transcriptome. The onset of steatosis in the liver was also evaluated histologically.

**Results:**

Our results indicate that transcriptional reprogramming directing immune cell engagement proceeds robustly, even in the absence of histologically detectable steatosis, following administration of high fat diet. In the liver transcriptomes of animals with steatosis, a preference for the engagement of regulators of T cell activation and myeloid leukocyte differentiation was also recorded as opposed to the steatosis-free livers at which non-specific lymphocytic activation was seen. As compared to controls, in the animals with steatosis, transcriptome was subjected to more widespread reorganization while in the animals without steatosis, reorganization was less extensive. Comparison of the steatosis and non-steatosis livers showed high retention of coordination suggesting that diet supersedes pathology in shaping the transcriptome’s profile.

**Conclusions:**

This highly versatile strategy suggests that the molecular changes inducing inflammation proceed robustly even before any evidence of steatohepatitis is recorded, either histologically or by differential expression analysis.

**Supplementary Information:**

The online version contains supplementary material available at 10.1186/s12864-021-07784-y.

## Introduction

Non-alcoholic steatohepatitis (NASH) develops in livers that have accumulated histopathological changes associated with hepatic steatosis and are reflected to the differential expression of genes linked to the induction of inflammation [1–3]. During disease progression extensive transcriptional reprogramming occurs that underscores its different stages. This multistage process can be recapitulated with relatively high accuracy in animal models receiving special diets, alone or combined with other stimuli triggering liver injury [4]. Among them, outbred models may be of special value since they can mimic the different courses of disease progression in human patients at which steatosis develops stochastically [5].

Essential for the molecular characterization of different subtypes of liver disease is differential expression which points to specific transcripts that are enriched or depleted at different disease stages [6–8]. Such quantitative changes in expression, usually illuminate full-fledged pathology while subtle alterations, despite their potential significance may remain unnoticeable. To overcome these limitations, we have proposed a novel strategy that instead of the expression levels of individual transcripts takes into consideration the degree of correlation of expression of each transcript with every other transcript in the transcriptome. Evaluation of this coordination profile of the whole liver transcriptome at different disease stages, may provide hints regarding the underlying molecular changes that conventional, differential expression analysis cannot. Such changes in coordination profile appeared relevant in characterizing different liver pathology stages by focusing on the unfolded protein response [9–11]. Furthermore, such analysis applied to the most highly expressed genes in the transcriptome was shown capable of illustrating changes in patients with frailty syndrome [12]. More recently, application of this strategy to the transcriptome of deer mice (Peromyscus) indicated changes in gene expression profiles and associated biological processes that occur in the brain during aging in different species [13].

In the present study we used outbred deer mice (*Peromyscus maniculatus*) as a model to evaluate how the liver transcriptome is collectively reorganized in specimens with or without steatosis. Our studies were based on our earlier findings indicating that upon high fat diet administration (HFD) *P. maniculatus* do not develop steatohepatitis, but a subset, about 50%, develops hepatic steatosis [5]. This provides an animal model at which disease development can recapitulate human genetically diverse populations at which disease susceptibility varies considerably among individuals [14].

The premise of the present analysis is that genes belonging to the same transcriptional networks are co-expressed, but when pathology emerges the profile of co-expression is collectively changed [15–21]. To address the coordination profile, we calculated the composite correlation for each gene in the transcriptome with every other gene and compared it in the controls, the animals that received HFD but did not develop steatosis and the animals that received HFD but developed steatosis. The results were coupled to gene ontology (GO) analyses [22] to reveal transcripts that more prominently abolished their coordination with the whole transcriptome. This analysis was recently applied to the brain transcriptome of deer mice and showed that during aging a loss of the perception of smell occurs in different species despite that de-coordination of the transcriptome exhibit interspecific variations [13]. Our present results, besides describing the overall coordination profile of the transcriptome at different conditions, also show that HFD triggers a robust induction of an inflammatory response, irrespectively of the onset of steatosis. This change was not apparent by conventional analyses of the differentially expressed transcripts. Furthermore, it showed that what differentiates the liver transcriptomes with and without steatosis is the preference of the former for T cell activation, myeloid leukocyte differentiation and engagement of genes involved in cell cycle regulation.

## Results

### Variable response to HFD in outbred deer mice

**Fig. 1:**
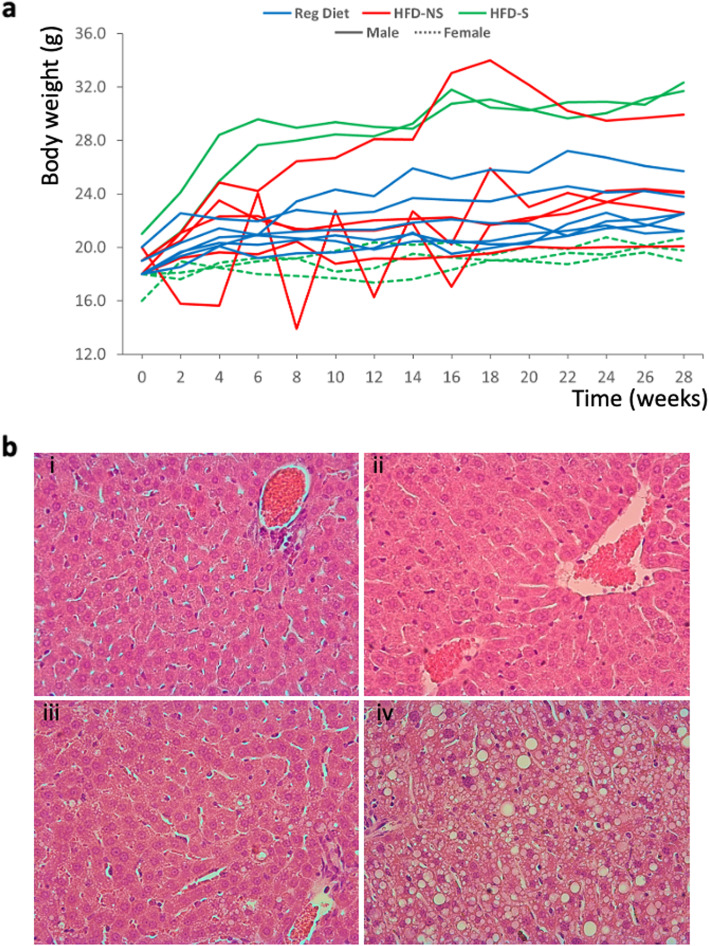
Response of deer mice *(P. maniculatus*) to HFD. **a.** Body weight in genetically diverse *P. maniculatus* after administration of regular diet or HFD. Sex, diet and development of steatosis are indicated. Highly variable response was recorded that was not associated with any of the parameters recorded. **b**. Histopathological appearance of liver sections (H&E) from animals that received regular diet (i) or HFD (ii) but did not develop steatosis or received HFD and developed moderate (iii) or severe (iv) steatosis

Earlier studies showed that *P. maniculatus* exhibits variable response to HFD. In the present study, a panel of 3–4 months old, outbred deer mice (*P. maniculatus*) received HFD for about 6 months (*n* = 10). Six animals received regular diet. Body weight was increased in the animals that received the HFD but body weight gain remained highly variable, consistently with the genetically diverse nature of the experimental animals (Fig. 1a). Histology revealed the presence of steatosis in 5 out of 10 animals that received HFD (Fig. 1b). No evidence of fibrosis, ballooning degeneration or lobular inflammation was recorded [23–25], suggesting that under these conditions the disease has not progressed to more advanced stages of non-alcoholic liver steatohepatitis (NASH) (Fig. 1b).

### Distinct profile of expression coordination in livers with or without steatosis

RNAseq was performed in the liver of *P. maniculatus* that received regular diet or HFD and results have been deposited to NCBI (GSE146846). To test how the transcriptome in each group is coordinated at these conditions we calculated the composite correlation (Pearson’s composite, Pc) index as follows: Initially we calculated the correlation coefficient for each gene with every other expressed gene in the transcriptome, in all 3 pairwise comparisons being control vs steatosis (C vs S), control vs non-steatosis (C vs NS) and steatosis vs non steatosis (S vs NS) (Supplementary Tables 1, 2, 3). The analysis was performed by using a code in R language we have developed and involved 13,340 transcripts in the NS vs C comparisons, 13,434 transcripts in the S vs C comparisons and 12,041 transcripts in the S vs NS comparisons. In all cases, the expression of each of these transcripts was evaluated versus all other transcripts in the transcriptome of the experimental specimens. Pc of each transcript reflects the composite correlation coefficient of all correlation coefficients that were calculated as described above, for each given transcript, in the 3 pairwise comparisons (C vs S, C vs NS, and S vs NS). Therefore, high Pc values indicate that coordination is retained for the given comparison, while lower Pc values indicate loss of coordination. Conversely, negative Pc values show that the profile of coordination is inversed in a manner according to which positive correlation with the transcriptome in one experimental condition is inversed to negative in the other, and vice versa.

**Fig. 2:**
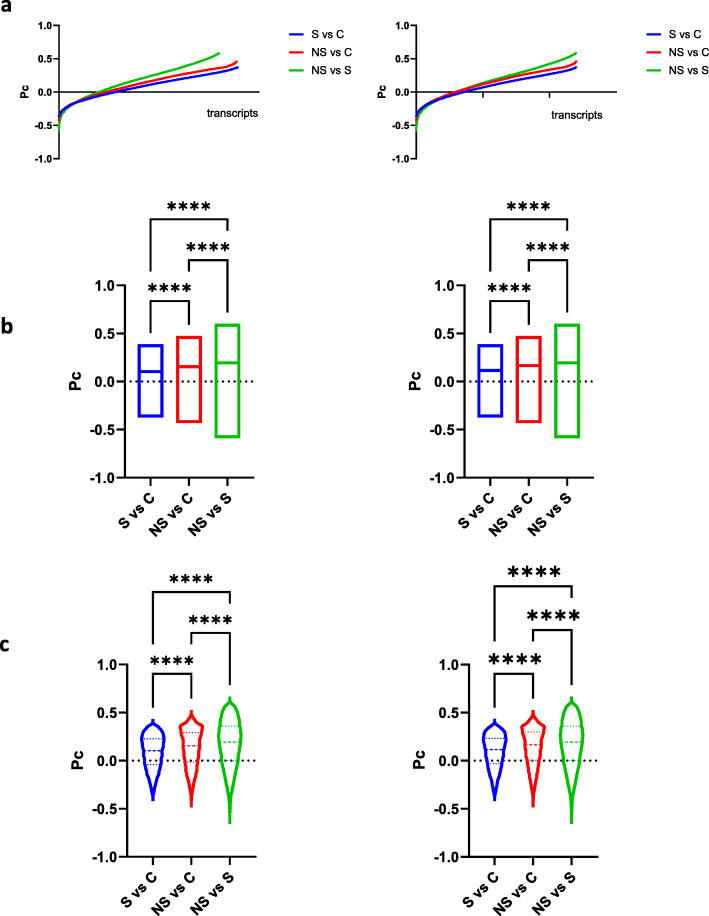
Pc calculation for the liver transcriptome of *P. maniculatus* fed with regular diet (C) or HFD and developed (S) or did not develop (NS) steatosis. Scatter plots of Pc versus transcripts are shown in (**a**), barr plots showing the median values are shown in (**b**), and box and violin plots depicting Pc distribution are shown in (**c**). In the left panel results from all genes surveyed are shown while in the right panel only results from common genes in all 3 pairwise comparisons are shown. ****, *P* < 0.00001 (Ordinary one-way ANOVA, Tukey’s multiple comparisons test)

As shown in Fig. 2a, in all three groups, most of the transcripts showed positive Pc values, which indicates that coordination is retained between the animals with or without steatosis, for most of the genes. Higher Pc values (average Pc = 0.17) were seen in the S vs NS groups suggesting that most genes retained their coordination upon HFD administration and irrespectively of the development of steatosis (Fig. 1b,c). Conversely, lowest Pc (=0.086) was seen in the comparison between S and C suggesting extensive transcriptional reprogramming. In the comparison between NS and C, average Pc had intermediate magnitude (Pc = 0.13). All differences were statistically significant (ANOVA, *P* < 0.0001). Similar were the findings when instead of the whole transcriptome only genes common in the 3 groups were evaluated suggesting that the findings do not reflect a bias towards transcripts that are present only in some experimental groups (Fig. 2).

These results suggest that during HFD administration, extensive reprogramming of the transcriptome occurs, which is more pronounced in the livers that develop steatosis as compared to those that did not. The differences in the transcriptomic profile between the livers that did and those that did not develop steatosis at HFD, were more modest (Pc = 0.17). Thus, special diet such as HFD induces more changes in the transcriptome than the pathology (steatosis) per se.

### Gene ontology analyses reveal engagement of inflammation by HFD

To obtain insights regarding the biological processes that are enriched for the transcripts exhibiting the most pronounced changes in Pc values in the 3 comparison groups we utilized the publicly available Gene Ontology Platform (Gene Ontology http://geneontology.org/). For this analysis, the Pc values were sorted for each group in descending order and the genes exhibiting Pc < − 0.2 were analyzed (Suppl Table 4). The results for the top 10 processes are shown in Table 1 and were derived by using 752 genes, 700 genes and 854 genes for the S vs C, the NS vs C and the S vs NS genes respectively. Both comparisons involving administration of HFD (S and NS) vs C exhibited an enrichment for processes associated with a proinflammatory response. Thus, robust transcriptional reprogramming, consistent with the induction of inflammation, occurs irrespectively of steatosis and despite that no histopathological evidence of inflammation was seen. Comparison between the S vs NS specimens revealed that the most prominent processes were associated with cell cycle regulation.

**Table 1:** Gene Ontology analysis based on Pc data. Genes having Pc < − 0.2 were considered. For cumulative Pc analysis, the 3 individual Pc were added and the genes within the 5th percentile of those with higher cumulative Pc were considered

GO biological process complete	Fold Enrichment	raw ***P***-value	FDR
**Steatosis vs Control**			
positive regulation of chemokine (C-X-C motif) ligand 2 production (GO:2000343)	11.63	2.14E-04	4.27E-02
myeloid leukocyte activation (GO:0002274)	3.33	6.62E-05	2.01E-02
myeloid leukocyte differentiation (GO:0002573)	3.28	2.14E-04	4.32E-02
positive regulation of T cell activation (GO:0050870)	3.21	3.26E-06	2.71E-03
cytokine production (GO:0001816)	3.08	9.61E-05	2.40E-02
positive regulation of leukocyte cell-cell adhesion (GO:1903039)	3.02	5.30E-06	2.98E-03
positive regulation of leukocyte proliferation (GO:0070665)	2.94	2.50E-04	4.75E-02
cell activation involved in immune response (GO:0002263)	2.7	2.75E-04	4.97E-02
regulation of T cell activation (GO:0050863)	2.69	2.12E-06	1.97E-03
regulation of leukocyte cell-cell adhesion (GO:1903037)	2.67	3.55E-06	2.66E-03
**Non steatosis vs Control**			
neuronal action potential propagation (GO:0019227)	13.51	1.17E-04	4.49E-02
action potential propagation (GO:0098870)	13.51	1.17E-04	4.38E-02
plasma membrane organization (GO:0007009)	3.82	8.77E-05	3.63E-02
lymphocyte differentiation (GO:0030098)	2.66	1.60E-05	1.09E-02
mononuclear cell differentiation (GO:1903131)	2.46	4.78E-05	2.28E-02
leukocyte differentiation (GO:0002521)	2.36	3.51E-05	1.84E-02
lymphocyte activation (GO:0046649)	2.35	9.87E-06	7.77E-03
leukocyte activation (GO:0045321)	2.22	5.65E-06	5.56E-03
hemopoiesis (GO:0030097)	2.1	3.73E-06	5.33E-03
hematopoietic or lymphoid organ development (GO:0048534)	1.97	1.44E-05	1.08E-02
**Steatosis vs Non-Steatosis**			
mitotic G2/M transition checkpoint (GO:0044818)	6	6.01E-05	2.70E-02
negative regulation of G2/M transition of mitotic cell cycle (GO:0010972)	5.24	6.31E-05	2.68E-02
negative regulation of cell cycle G2/M phase transition (GO:1902750)	4.68	6.85E-05	2.77E-02
regulation of G2/M transition of mitotic cell cycle (GO:0010389)	4.64	1.79E-06	1.76E-03
regulation of cell cycle G2/M phase transition (GO:1902749)	3.9	1.26E-05	8.61E-03
negative regulation of mitotic cell cycle phase transition (GO:1901991)	3.15	7.98E-05	3.14E-02
negative regulation of cell cycle phase transition (GO:1901988)	3.12	3.47E-05	1.89E-02
regulation of mitotic cell cycle phase transition (GO:1901990)	2.79	1.79E-06	1.66E-03
regulation of cell cycle phase transition (GO:1901987)	2.56	8.13E-06	6.40E-03
mitotic cell cycle (GO:0000278)	2.06	1.73E-05	1.09E-02
**Pc (cumulative) with top 5% of genes (600)**			
mammary gland epithelium development (GO:0061180)	5.14	1.34E-04	4.91E-02
cellular modified amino acid metabolic process (GO:0006575)	3.59	2.51E-05	1.36E-02
sulfur compound metabolic process (GO:0006790)	2.95	9.59E-06	5.59E-03
glycerophospholipid metabolic process (GO:0006650)	2.76	1.29E-04	4.85E-02
phospholipid metabolic process (GO:0006644)	2.59	6.53E-05	2.94E-02
cellular lipid metabolic process (GO:0044255)	2.4	5.38E-09	9.41E-06
organophosphate metabolic process (GO:0019637)	2.37	1.09E-07	1.07E-04
monocarboxylic acid metabolic process (GO:0032787)	2.26	6.99E-05	3.06E-02
carboxylic acid metabolic process (GO:0019752)	2.19	2.63E-06	1.80E-03
organic acid metabolic process (GO:0006082)	2.18	1.16E-06	9.65E-04

In order to identify genes that collectively exhibit changes in their transcriptomic profile across the different groups, we calculated a cumulative Pc index by adding the 3 independent Pc indices for the genes that were common between the 3 individual pairwise comparisons. Then we sorted the genes in descending order according to their cumulative Pc and selected the top 5% which corresponded to about 600 genes for GO analysis (Table 1 and Suppl Table 5). Not surprisingly, GO analysis indicated that processes associated with metabolism were more prominently enriched.

### Differential expression only reveals a fraction of processes linked to steatosis

**Fig. 3:**
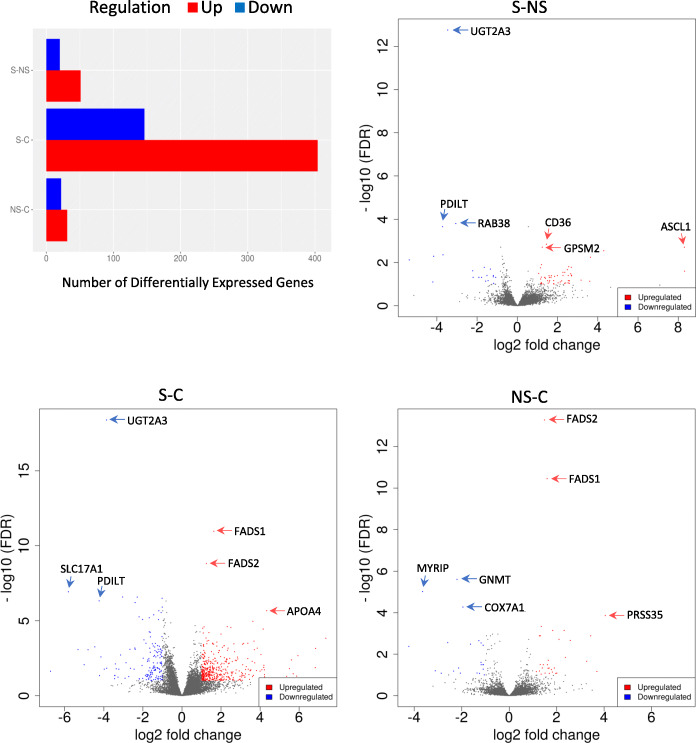
Number of differentially expressed genes, the volcano plots and the top 3 upregulated and down regulated genes in all 3 pairwise comparisons. FDR cutoff is 0.1 and minimum fold change is 2

To appreciate the discovery power of the proposed transcriptome coordination strategy, we also performed conventional differential expression and GO analysis (Suppl Table 6). For this analysis the iDEP online platform was used by using FDR cut-off 0.1 and minimum fold change 2. As shown in Table 2 the results were not highly consistent and informative in the different groups and suggested enrichment of processes associated with lipid metabolism in the NS vs C, while in the S vs C comparison, processes associated with cytokinesis, mitotic spindle formation and epithelial to mesenchymal transition. In the S vs NS comparison, the most prominent processes enriched were related to apoptosis and regeneration. The number of differentially expressed genes and the top 3 upregulated and downregulated transcripts in each comparison are shown in Fig. 3.

**Table 2:** Gene Ontology analysis based on differential expression. The results for the top 10 processes are shown in the table

GO biological process complete	Fold Enrichment	raw ***P***-value	FDR
**Steatosis vs Control**			
actomyosin contractile ring organization (GO:0044837)	25.52	6.19E-04	2.21E-02
actomyosin contractile ring assembly (GO:0000915)	25.52	6.19E-04	2.20E-02
assembly of actomyosin apparatus involved in cytokinesis (GO:0000912)	25.52	6.19E-04	2.20E-02
regulation of postsynaptic cytosolic calcium ion concentration (GO:0099566)	25.52	6.19E-04	2.19E-02
positive regulation of epithelial to mesenchymal transition involved in endocardial cushion formation (GO:1905007)	25.52	6.19E-04	2.19E-02
negative regulation of lipoprotein lipase activity (GO:0051005)	24.3	7.99E-05	4.58E-03
glomerular visceral epithelial cell migration (GO:0090521)	21.26	9.13E-04	2.88E-02
mitotic spindle midzone assembly (GO:0051256)	21.26	9.13E-04	2.88E-02
mitotic spindle elongation (GO:0000022)	21.26	9.13E-04	2.87E-02
regulation of epithelial to mesenchymal transition involved in endocardial cushion formation (GO:1905005)	21.26	9.13E-04	2.86E-02
**Non steatosis vs Control**			
regulation of intestinal cholesterol absorption (GO:0030300)	> 100	2.85E-06	8.98E-03
regulation of intestinal lipid absorption (GO:1904729)	> 100	3.56E-06	8.01E-03
regulation of intestinal absorption (GO:1904478)	91.62	7.54E-06	1.48E-02
positive regulation of fatty acid biosynthetic process (GO:0045723)	56.38	2.80E-05	3.15E-02
positive regulation of triglyceride metabolic process (GO:0090208)	50.55	3.79E-05	3.73E-02
positive regulation of lipid catabolic process (GO:0050996)	47.29	4.56E-05	4.22E-02
positive regulation of fatty acid metabolic process (GO:0045923)	42.49	3.26E-06	8.57E-03
regulation of fatty acid metabolic process (GO:0019217)	20.57	4.96E-05	4.34E-02
steroid biosynthetic process (GO:0006694)	19.94	5.57E-05	4.62E-02
monocarboxylic acid biosynthetic process (GO:0072330)	16.4	1.52E-05	2.18E-02
**Steatosis vs Non-Steatosis**			
positive regulation of phagocytosis, engulfment (GO:0060100)	16	3	0.05
positive regulation of membrane invagination (GO:1905155)	16	3	0.05
animal organ regeneration (GO:0031100)	27	4	0.09
regeneration (GO:0031099)	101	5	0.34
regulation of cell death (GO:0010941)	1667	20	5.53
regulation of programmed cell death (GO:0043067)	1504	18	4.99
regulation of apoptotic process (GO:0042981)	1468	17	4.87
regulation of cell population proliferation (GO:0042127)	1691	18	5.61
cellular response to organic substance (GO:0071310)	1816	18	6.03
response to organic substance (GO:0010033)	2529	22	8.4

### Immune cell markers do not exhibit differential expression in the livers of deer mice receiving HFD

**Fig. 4:**
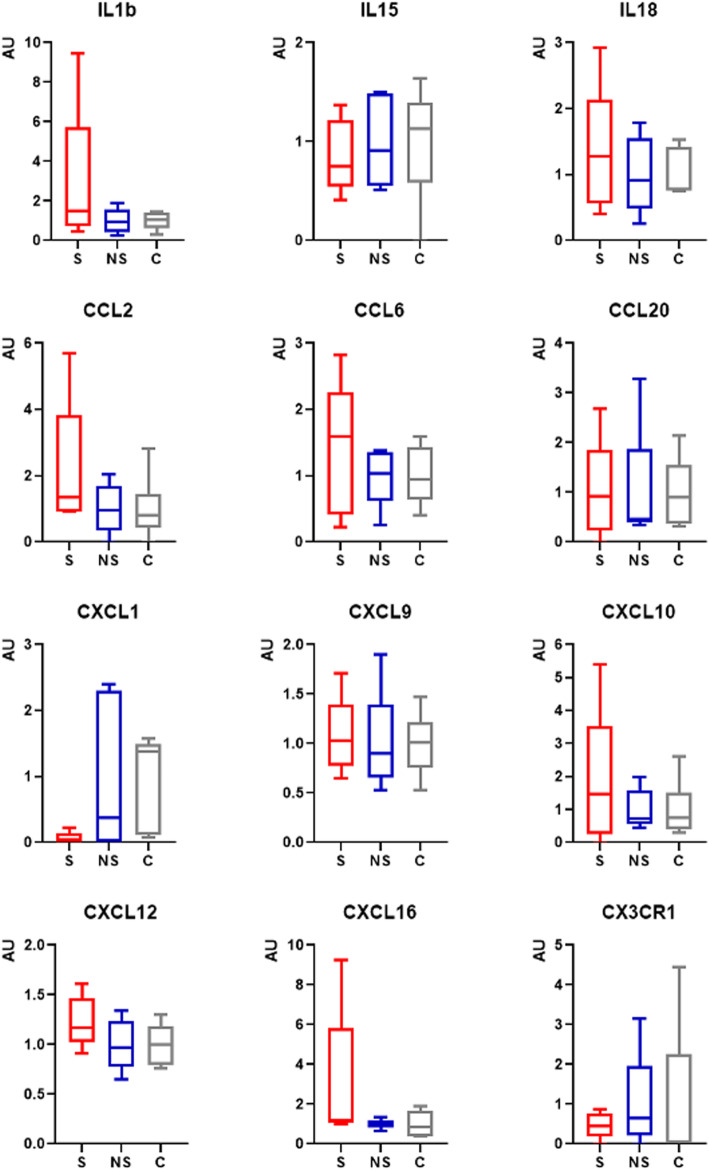
Expression of cytokine and chemokine genes in the livers of control (C), and animals that received HFD and developed steatosis (S) or did not develop steatosis (NS). Data were extracted from the RNA-Seq read counts and normalized with TBP (TATA-Box Binding Protein) expression. Statistical analysis was performed by ordinary one-way ANOVA applying Tukey’s multiple comparisons test, and no results were significant (*n* = 6 for C, and *n* = 5 for each of S and NS) .

Despite that immune cell activation was not detectable by the conventional differential expression analysis it remains plausible that individual mediators of the proinflammatory response are differentially expressed. We tested this by evaluating the expression of a panel of immune cell markers including IL1b, IL15, IL18, CCL2, CCL6, CCL20, CXCL1, CXCL9, CXCL10, CXCL12, CXCL16, and CX3CR1 but none showed differential expression levels between the experimental groups (Fig. 4, not significant, Ordinary one-way ANOVA, Tukey’s multiple comparisons test).

## Discussion

The assessment of differential expression is an important indicator of genes associated with pathology however its value can be limited when genetically diverse specimens are analyzed. Genes highly relevant to disease may remain masked if the variation in gene expression between individuals reduces the statistical power of differential expression studies. For example, in hepatic steatosis, despite the established role of endoplasmic reticulum stress in disease development, genes associated with the unfolded protein response are not usually detected by differential expression analysis [6]. Such role though can be revealed when their coordination with the whole transcriptome is examined [9]. When the role of inflammation is studied in liver disease, it marks only its more advanced stages and is frequently dissociated from steatosis, especially in some animal models [26–29]. While the deregulation of pro-inflammatory cytokines is detected in benign steatosis in the absence of typical liver inflammation they are generally considered as the direct outcome of aberrant lipid metabolism, occasionally originating from visceral fat, are linked to insulin resistance and are not representative of an orchestrated inflammatory response occurring in the liver [30–32].

By using a novel, unbiased whole transcriptome analysis, that relies on the extent of expression of all transcripts in livers from outbred rodents that developed or not steatosis after HFD administration, we were able to show that both in the specimens that did not show pathology and those that exhibited steatosis, a robust engagement of proinflammatory processes occurred. What however differentiated the two entities was the engagement of T cell activation and myeloid leukocyte differentiation processes that was detected only in the fatty livers and could be due to the presence of genetic modifiers that differentiates the responses recorded between the groups. Conventional differential expression analysis that focuses on transcripts exhibiting quantitative differences in the experimental groups, failed to reveal evidence of immune cell activation. This was further supported by the lack of significant changes in the expression of a panel of established mediators of inflammation in liver disease. Probably this limitation is related to the genetically diverse nature of the specimens in combination with the fact that such changes may be below the thresholds of significance of such analysis. Furthermore, it may indicate that in this animal model, under the present conditions and at this stage of disease development, only some transcriptional reprogramming had occurred that has primed cells for the mobilization of the immune cells. The latter, however, has not occurred substantially, as yet, because differential expression was not implemented. Such priming for transcriptional reprogramming may have committed the liver tissue into a permissive state at which pro-inflammatory changes occurred, even prior to the engagement of specific markers that are diagnostic for immune cell engagement. Coordination analysis, especially at the whole transcriptome level, leverages such diversity in gene expression among individual specimens and can extract meaningful information even when subtle changes occurred. To that end, it is conceivable that prolonged high fat diet administration and/or diet richer in fat than the one presently used, may eventually cause histologically detectable changes consistent with hepatic inflammation and steatosis in the animals that received HFD but did not develop steatosis so far. Consistently with this notion, it is plausible that application of this analysis in human specimens may be capable of revealing changes prior to the manifestation of specific histological or molecular alterations.

This coordination analysis also indicated in pairwise comparisons that a major difference of the livers with and without steatosis, as compared to the controls is that in those with steatosis, the transcriptome underwent more extensive reorganization compared to those without. Comparison though of the two, exhibited the higher retention in the profile of coordination. This suggests that diet supersedes pathology in shaping the profile of the transcriptome. From a different perspective this observation implies that for the emergence of pathology, such as steatosis, a more restricted roster of changes in gene expression is sufficient, while more global changes, likely imply the operation of homeostatic mechanisms that maintain normal cellular function.

Conventional differential expression analysis of gene expression was only able to reveal inconsistent changes between the experimental groups, among which the most prominent were related to lipid metabolism in NS vs C, cytokinesis, mitotic spindle formation and epithelial to mesenchymal transition in S vs C, and processes related to apoptosis and regeneration in S vs NS comparison.

Collectively, these results suggest that inflammatory engagement is robustly triggered by HFD even before inflammation is detectable in the histopathological analysis or by the differential expression studies, and illustrate the power of the proposed gene coordination approach to reveal changes that conventional strategies cannot.

## Materials and methods

### Animals

Deer mice, *P. maniculatus* were obtained from the Peromyscus Genetic Stock Center (PGSC) of the University of South Carolina (UofSC), Columbia, SC (RRID:SCR_002769). Deer mice, *P. maniculatus bairdii* (BW Stock), were bred as a closed colony in captivity, since 1948 and descended from 40 ancestors wild-caught near Ann Arbor, Michigan [14]. Deer mice were fed either a regular chow diet (*n* = 6) or a high fat diet (HFD, 58 kcal% fat and sucrose, Research Diets D12331) (*n* = 10) for 28 weeks, starting at 3–4 months of age. A larger number of animals received HFD to obtain cohorts with and without steatosis [5]. Body weight was measured every 2 weeks. Animals were then sacrificed using isoflurane as an anesthetic followed by cervical dislocation, and the livers were collected. All animal procedures were approved by the Institutional Animal Care and Use Committee (IACUC) and the Department of Health and Human Services, Office of Laboratory Animal Welfare, University of South Carolina (Approval No. 2349–101,211-041917). All methods were carried out in accordance with relevant guidelines and regulations. The study was carried out in compliance with the ARRIVE guidelines.

### RNA sequencing

RNA and library preparation, sequencing, and postprocessing of the raw data and data analysis were performed by the USC CTT COBRE Functional Genomics Core. RNAs were extracted with a Qiagen RNeasy Plus Mini kit as per manufacturer’s recommendations (Qiagen, Valencia, CA). RNA integrity was assessed using the Agilent Bioanalyzer and samples had a quality score ≥ 8.6. RNA libraries were prepared using established protocol with NEBNext Ultra II Directional RNA Library Prep Kit for Illumina (NEB, Lynn, MA). Each library was made with one of the TruSeq barcode index sequences and pooled together into one sample to be sequenced on the HiSeq 2x150bp pair-ended sequencing platform (Genewiz). Sequences were aligned to the *P. maniculatus* genome (HU_Pman_2.1 (GCA_003704035.1)) in ensembl.org using STAR v2.7.2 [33]. Reads were counted using the featureCounts function of the Subreads package [34] using Ensembl GTF and summarized at exon, transcript, or gene level. Only reads that were mapped uniquely to the genome were used. The differentially expressed gene analysis was conducted with iDEP.91 (iDEP Platform http://bioinformatics.sdstate.edu/idep/) [35]. The criteria used for this analysis were FDR with cut-off 0.1 and minimum fold change in gene expression of 2.

### Histology

Upon termination of the study the liver of the animals was removed, fixed in 10% neutral buffered formalin and paraffin embedded. The livers were stained with H&E and were histologically evaluated. Histological examination of the liver specimens was performed blindly for the presence of hepatic steatosis according to the scoring system designed by the Pathology Committee of the NASH Clinical Research Network, which addresses the full spectrum of lesions of NAFLD [36]. Images shown were obtained by a Leica ICC50 HD.

### Coordination analysis

The correlation coefficients (R, Person’s) values of each gene against all other genes in the transcriptome were calculated in specimens of steatosis vs nonsteatosis, steatosis vs control and nonsteatosis vs control, respectively. The composite correlation (Pc) index was evaluated by calculating the Person’s R of all R coefficients, of each gene in each group combination. This transformation resulted in the development of a unique Pc value corresponding to each of these genes. This reflects the extent by which coordination with the whole transcriptome changes in the corresponding gene. All calculations were conducted with R 3.6.3.

### Statistical analysis

For differential expression results were analyzed by using the iDEP platform default values (FDR cut-off = 0.1 and minimum fold change = 2). For GO analyses Fisher test was used by applying FDR correction (details in corresponding supplementary Tables). For correlation studies, R value from Pearson’s correlation was calculated. Comparisons between groups for both Pc values and differential expression of qPCR results were performed by ordinary one-way ANOVA and by applying Tukey’s multiple comparisons test. In all case results were considered significant when *P* < 0.05.

## Supplementary Information

**Additional file 1: Supplementary Table 1.** Pc values for each gene derived from the comparison of NS and C groups.

**Additional file 2: Supplementary Table 2.** Pc values for each gene derived from the comparison of S and C groups.

**Additional file 3: Supplementary Table 3.** Pc values for each gene derived from the comparison of NS and S groups.

**Additional file 4: Supplementary Table 4.** GO analysis results for genes exhibiting Pc < − 0.2 in all experimental group comparisons.

**Additional file 5: Supplementary Table 5.** Cummulative Pc and corresponding GO analysis for 5% of transcripts with higher Pc.

**Additional file 6: Supplementary Table 6.** GO analysis for differentially expressed genes in all experimental groups comparisons.

## Data Availability

Deer mice are available from the Peromyscus Genetic Stock Center. Data were deposited to NCBI. Accession No. GSE146846. Permanent link: https://www.ncbi.nlm.nih.gov/geo/query/acc.cgi?acc=GSE146846
